# Comprehensive Assessment of the Association of *WNK4* Polymorphisms with Hypertension: Evidence from a Meta-Analysis

**DOI:** 10.1038/srep06507

**Published:** 2014-09-30

**Authors:** Xiao-gang Guo, Jie Ding, Hui Xu, Tian-ming Xuan, Wei-quan Jin, Xiang Yin, Yun-peng Shang, Fu-rong Zhang, Jian-hua Zhu, Liang-rong Zheng

**Affiliations:** 1 Department of Cardiology, the First Affiliated Hospital, School of Medicine, Zhejiang University, Hangzhou 310003, China; 2 Xiuzhou District, Gaozhao Street Community Health Service Center, Jiaxing 314031, China

## Abstract

The relationship between with-no-lysine [K] kinase 4 (*WNK4*) gene polymorphisms and hypertension has been widely investigated, However, the studies yielded contradictory results. To evaluate these inconclusive findings comprehensively, we therefore performed a meta-analysis. Ten articles encompassing 16 independent case-control studies with 6089 hypertensive cases and 4881 normotensive controls were selected for this meta-analysis. Four *WNK4* gene polymorphisms were identified (G1155942T, G1156666A, T1155547C, and C6749T). The results showed statistically significant associations of G1155942T polymorphism (allelic genetic model: odds ration or OR = 1.62, 95% confidence interval or CI: 1.11–2.38, *P* = 0.01; dominant model: OR = 1.85, 95% CI: 1.07–3.19, *P* = 0.03) and C6749T polymorphism (allele contrast: OR = 2.04, 95% CI: 1.60–2.59, *P*<0.01; dominant model: OR = 2.04, 95%CI: 1.59–2.62, *P*<0.01; and homozygous model: OR = 5.01, 95% CI: 1.29–19.54, *P* = 0.02) with hypertension risk. However, neither C1155547T nor G1156666A was associated significantly with hypertension susceptibility. In conclusion, this meta-analysis suggested that *WNK4* G1155942T and C6749T gene polymorphisms may contribute to the susceptibility and development of hypertension. Further well-designed studies with larger sample size are required to elucidate the association of *WNK4* gene multiple polymorphisms with hypertension risk.

Hypertension is a worldwide public health problem; its overall prevalence is approximately 30–45% in the general population with a steep increase with aging^1^. The number of adults with hypertension are projected to be increased by 60% about 1.56 billion in 2025^2^. Increased blood pressure is considered to be a major risk factor for cardiovascular, cerebrovascular and renal disease and it is closely related to both cardiovascular and cerebrovascular endpoints, including heart failure, myocardial infarction, and stroke^3^.According to the epidemiological data, nearly 16.5% of all deaths worldwide are attributed to hypertension, including 51% of deaths by strokes and 45% of deaths by coronary heart disease^4^. As the major controlled risk factor, prevention and treatment of hypertension might be of great significance to restrain the morbidity and mortality from cardio-cerebrovascular disease. However, the pathogenesis of hypertension is intricate and still has not been thoroughly understood. Epidemiological studies have suggested that 30–50% of blood pressure (BP) variation within a population is determined by the genetic origin^5^, however, the genes underlying the BP regulation have not been clearly elucidated.

BP is regulated by a complicated network, including vascular, renal, neuronal and endocrine mechanisms and is also influenced by environmental factors, genetic factors, and multifactorial interactions^6^. It is well established that sodium intake is positively associated, while potassium intake is inversely associated, with the hypertension prevalence^7^,^8^,^9^. Nonetheless, the BP response to dietary sodium and potassium intake seems to vary considerably among individuals because many individuals taking as much as 40 g of NaCl per day do not suffer from hypertension^7^.

Up to now, a serial of previously unrecognized kinases interacting each other in the distal nephron have been identified as playing important roles in sodium, potassium, and BP regulation. WNKs (with-no-lysine [K] kinases) are a novel family of serine-threonine protein kinases. They are different from other usual protein kinases in subdomain II of WNKs that lacks the common conserved catalytic lysine residue that is crucial for binding to ATP and protein phosphorylation^10^. There are four WNKs in mammalian^11^,^12^. Mutations of *WNK1* and *WNK4* are considered to be related with pseudohypoaldosteronism type II (PHA II), an autosomal dominant disease featuring hypertension, hyperkalemia, hyperchloremia and metablic acidosis^13^,^14^. Previous studies revealed that the pathphysiology of hypertension caused by mutations of *WNK1* and *WNK4* were involved in regulation of diverse ion transporters and channels of the distal nephron^15^,^16^,^17^,^18^. Mutations of *WNK1* and *WNK4* led to deregulated renal sodium absorption, potassium secretion, thereby giving rise to the PHA II phenotype^19^. Disease-causing mutations in *WNK1* are large deletions of the first intron that lead to the increased expression of wildtype *WNK1*, mutations in the *WNK4* gene are missense mutation and cluster within the highly conservative coding sequence outside the kinase domain^11^,^20^.

Subsequent numerous epidemiological studies were conducted to evaluate the relationship between *WNKs* gene variation and hypertension susceptibility, since Wilson FH *et al.* reported their potential relevance each other by studying a PHA II kindred^11^. Putku M *et al.* identified a significant association of *WNK1* AluYb8 insertion with BP variation in the Estonian HYPEST cohort study and confirmed this result by a meta-analysis of three independent European samples^21^. Consistently, similar effects of *WNK1* common and rare single nucleotide polymorphisms (SNPs) and haplotypes on BP have been found in both general population and hypertensives^21^,^22^,^23^,^24^. However, the association of *WNK4* polymorphisms with hypertension susceptibility is ambiguous on account of the multiple potentially related gene loci and inconsistent results have been reported in different studies^25^,^26^,^27^,^28^. In addition, no meta-analysis has yet been conducted to assess the relationship between *WNK4* polymorphisms and hypertension risk as we know. Therefore, in order to estimate the relationship between *WNK4* gene polymorphisms and hypertension, especially G1155942T, G1156666A, T1155547C, and C6749T, we performed this meta-analysis.

## Results

### Characteristics of the studies

**Figure 1:**
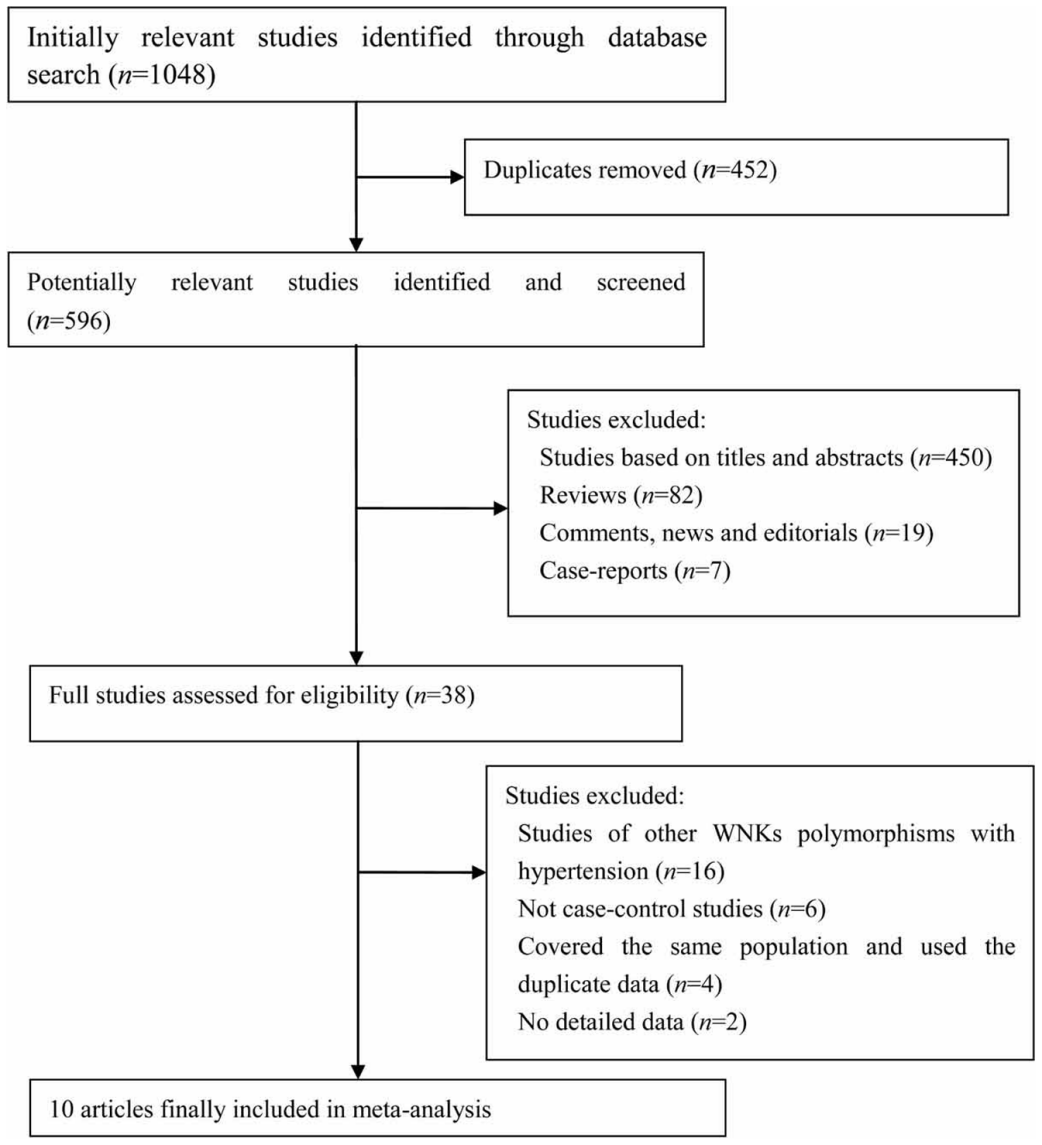
Flow diagram of the study selection procedure used for this meta-analysis of *WNK4* Polymorphisms and hypertension.

According to the inclusion criteria, 10 eligible studies^25^,^26^,^28^,^29^,^30^,^31^,^32^,^33^,^34^,^35^ were finally analyzed. The flow diagram of selection process of eligible studies is presented in Figure 1. Among all eligible studies, three articles by Erlich PM *et al.*^25^, Han Y *et al.*^29^, and Lu M *et al.*^32^ contained more than one study group, they were considered separately in the pooled analysis. Therefore, 16 independent case-control studies were finally collected in this meta-analysis to evaluate the relationship between the *WNK4* gene polymorphisms and hypertension including 6089 cases and 4881 controls. Main characteristics of these included studies are shown in Table 1, and the genotype distribution, allele frequencies and Hardy-Weinberg equilibrium (HWE) of controls are presented in Table 2. Genotype distributions of examined polymorphisms in controls were consistent with HWE for each study. By comparing the data of genotype distributions and allele frequencies, we found that one study by Lu M *et al.*^32^ which concerned with the G1155942T and C1155547T polymorphisms in a Chinese minority ethnic population showed quite different findings from the others. To avoid the influence of the overall results by each single study and get more cautious and credible results, we performed sensitive analysis by omitting each study sequentially as well. The main results of this meta-analysis of the relationship between the *WNK4* SNPs and hypertension risk are summarized in Table 3.

### Meta-analysis of *WNK4* C1155547T polymorphism and hypertension

**Figure 2:**
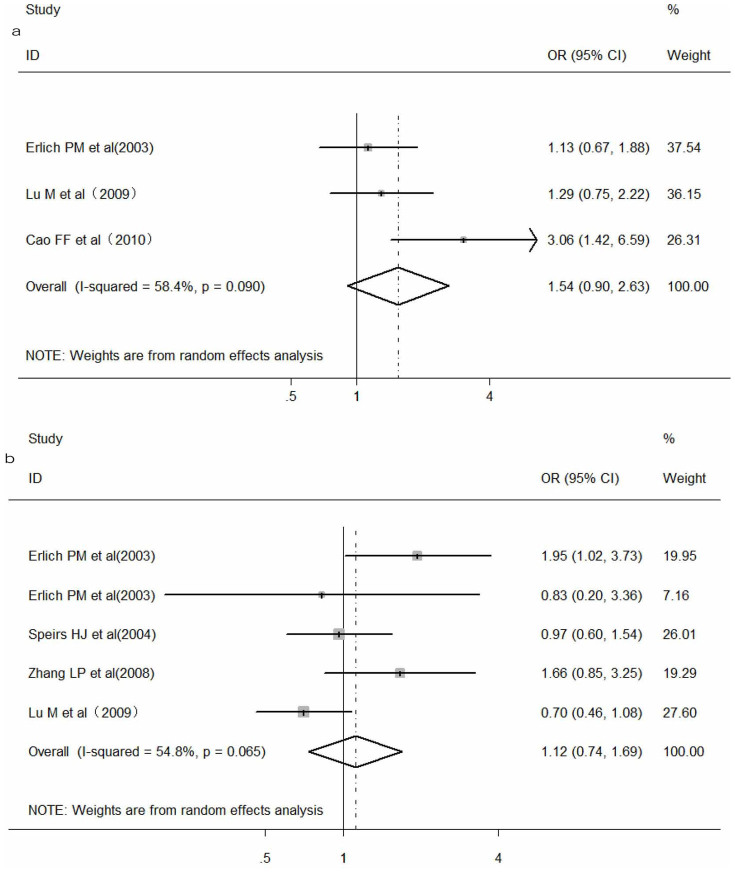
Meta-analysis of the association of *WNK4* C1155547T (a) and *WNK4* G1156666A (b) polymorphisms with hypertension under allele contrast respectively.

In total, three studies^25^,^31^,^32^ with 1096 cases and 874 controls were included in this meta-analysis to assess the relationship between C1155547T polymorphism and hypertension. No significant association was found between C1155547T polymorphism and the risk of hypertension under the allele contrast (odds ratio or OR = 1.54, 95% confidence interval or CI: 0.90–2.63, *P* = 0.11, Figure 2a), dominant model (OR = 1.70, 95% CI: 0.57–5.13, *P* = 0.35) and homozygous model (OR = 2.49, 95% CI: 0.57–10.78, *P* = 0.22). Because of the absence of wild homozygote (CC) in both case and control group, the study by Lu M *et al.*^32^ was excluded in the allele contrast and homozygous genetic model. Random-effects model was used in the allele contrast and dominant genetic model, for the heterogeneity between studies was significant (*P*_heterogeneity_ < 0.1, *I*^*2*^>50%), while fixed effects model was used in homozygous model.

### Meta-analysis of *WNK4* G1156666A polymorphism and hypertension

There were four articles^25^,^26^,^32^,^35^ including five studies focused on the correlation of *WNK4* G1156666A polymorphism with hypertension. Heterogeneity between studies was significant in allele contrast and dominant model (*P*_heterogeneity_ < 0.1, *I*^*2*^>50%), and thus we used a random-effects model for pooled analysis. One study by Erlich *et al.*^25^ was excluded in the homozygous model because of the absence of mutational homozygote (AA) in both case and control group. There was no evidence to support any significant association between *WNK4* G1156666A polymorphism and hypertension risk (allele genetic model: OR = 1.12, 95% CI: 0.74–1.69, *P* = 0.60, Figure 2b; dominant model: OR = 1.08, 95% CI: 0.68–1.71, *P* = 0.74; and homozygous model: OR = 3.40, 95% CI: 0.86–13.54, *P* = 0.08).

### Meta-analysis of *WNK4* G1155942T polymorphism and hypertension

**Figure 3:**
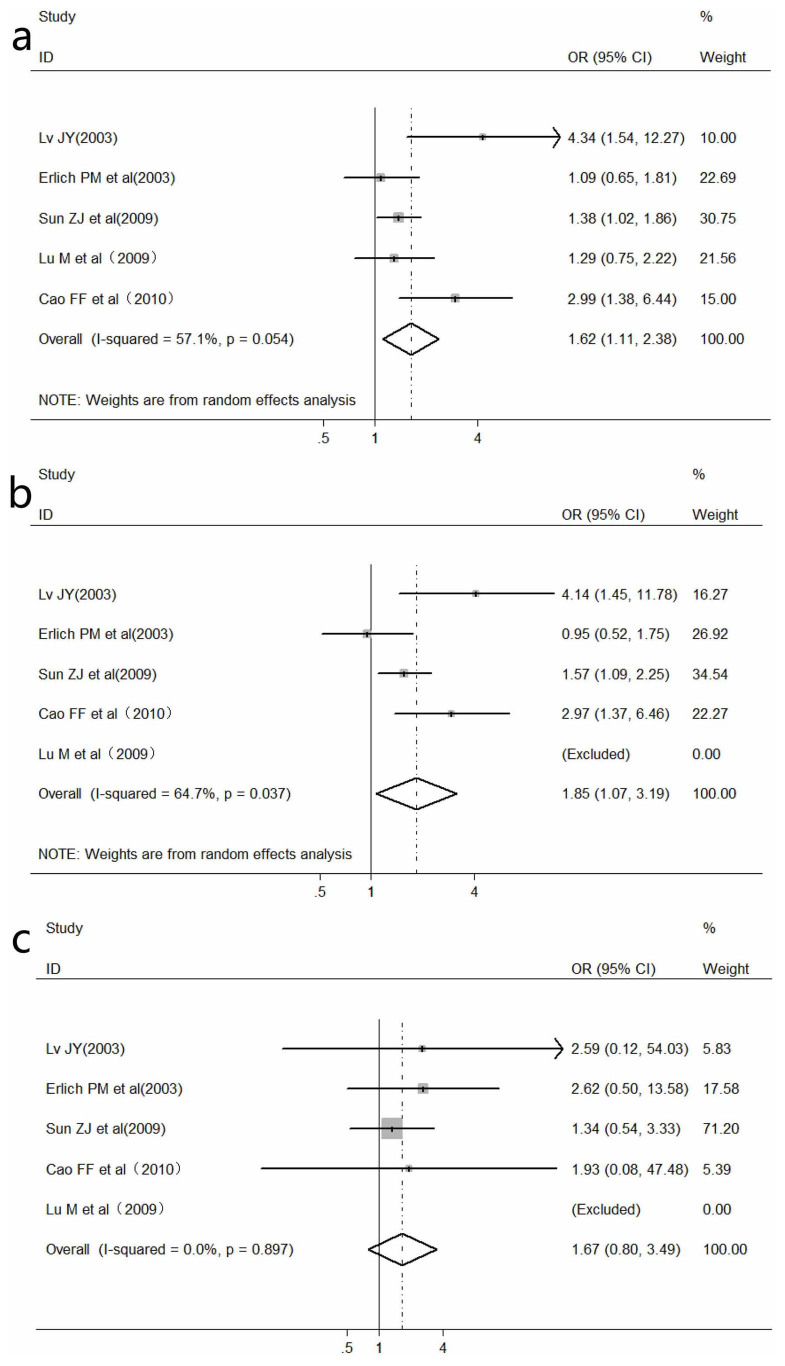
Meta-analysis of the association of *WNK4* G1155942T polymorphism with hypertension. a: under allele contrast; b: under dominant genetic model; c: under homozygote comparison.

To evaluate the association of *WNK4* G1155942T polymorphism with hypertension, five studies with 2260 cases and 1567 controls were pooled in this meta-analysis^25^,^28^,^30^,^32^,^34^. The study by Lu M *et al.*^32^ was excluded in pooled analysis in both dominant and homozygous genetic models, as a result of no existence of wild homozygote(GG) in both case and control group. Due to significant heterogeneity between the included studies, a random-effects model was used under allele contrast and dominant genetic model. Significant association between G1155942T polymorphism and hypertension was identified in both allele contrast (OR = 1.62, 95% CI: 1.11–2.38, *P* = 0.01) and dominant genetic model (OR = 1.85, 95% CI: 1.07–3.19, *P* = 0.03). However, no significant relation was found in homozygous genetic model (OR = 1.07, 95% CI: 0.80–3.49, *P* = 0.18) (Figure 3).

### Meta-analysis of *WNK4* C6749T polymorphism and hypertension

**Figure 4:**
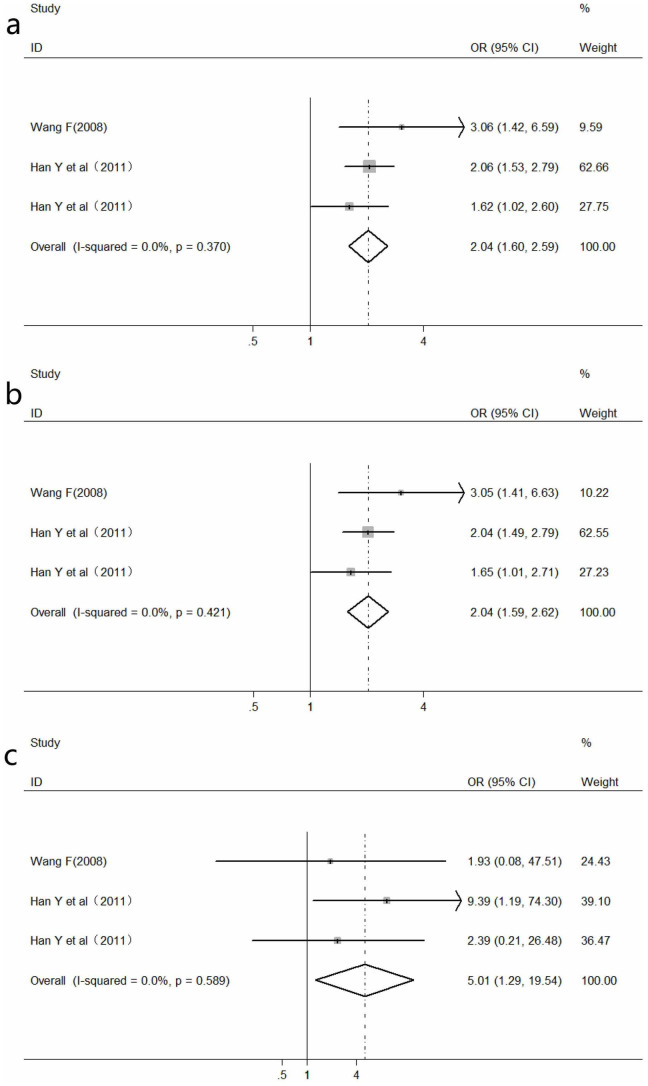
Meta-analysis of the association of *WNK4* C6749T polymorphism with hypertension. a: under allele contrast; b: under dominant genetic model; c: under homozygote comparison.

The association of *WNK4* C6749T polymorphism with hypertension was found to be significant in allele contrast (OR = 2.04, 95% CI/: 1.60–2.59, *P*<0.01), dominant genetic model (OR = 2.04, 95% CI: 1.59–2.62, *P*<0.01) and homozygous genetic model (OR = 5.01, 95% CI: 1.29–19.54, *P* = 0.02) (Figure 4). As the heterogeneity between studies was not significant (*P*_heterogeneity_ > 0.1, *I*^*2*^<50%), a fixed-effects model was used.

### Publication Bias

**Figure 5:**
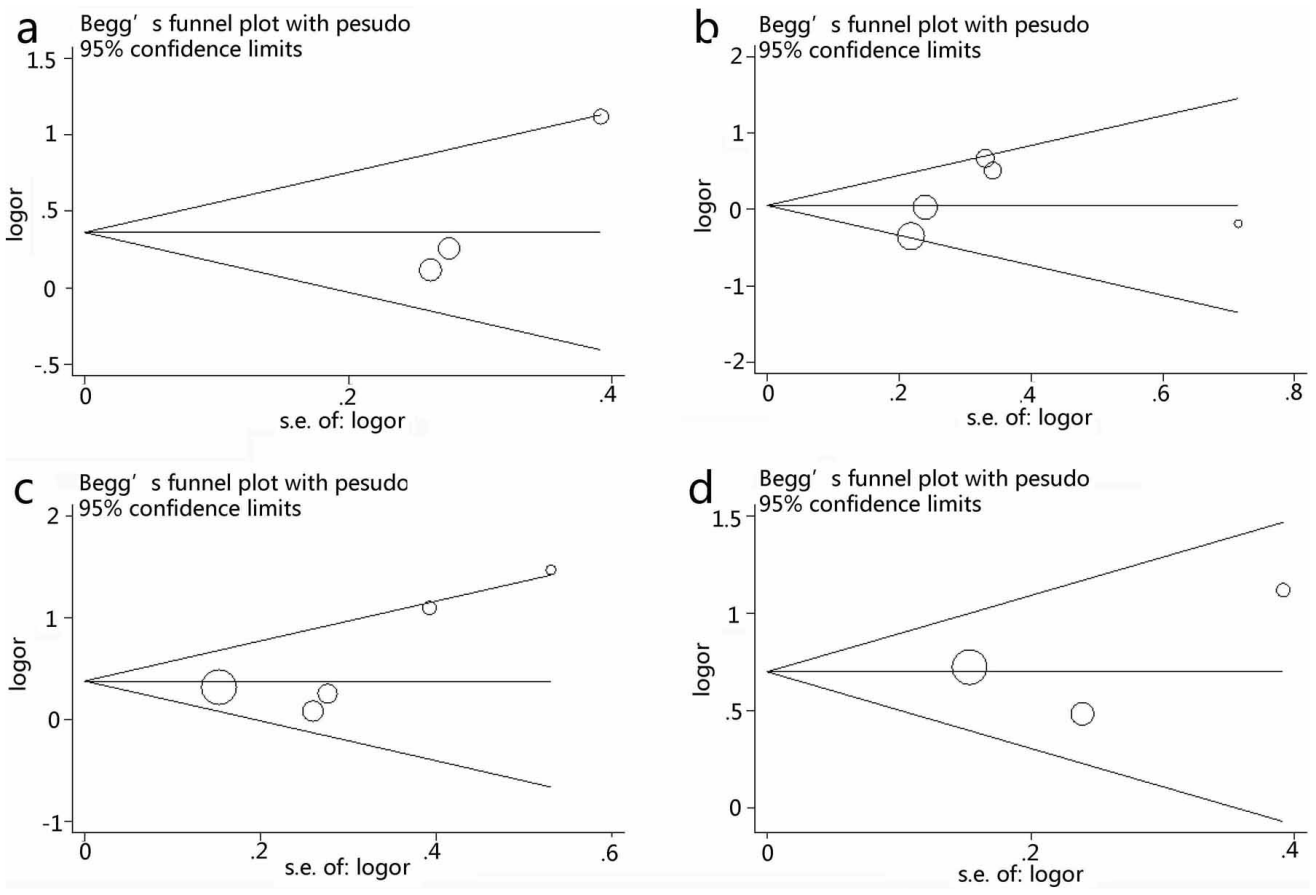
Begg's funnel plot of publication bias in the meta-analysis of the association of *WNK4* Polymorphisms with hypertension risk under allele genetic model. a: C1155547T and hypertension; b: G1156666A and hypertension; c: G1155942T and hypertension; d: C6749T and hypertension.

Both the Begg's test and Egger's regression test were performed to assess publication bias. The shapes of the funnel plots do not show obvious evidence of asymmetry (Figure 5). However, the *P*-value of Egger's test confirmed the existence of publication bias regarding the *WNK4* C1155547T polymorphism and hypertension under allele contrast (*P* = 0.02). No statistical significance of publication bias was observed by Egger's test to G1156666A (*P* = 0.50), G1155942T (*P* = 0.18) and C6749T (*P* = 0.75) polymorphisms under allele contrast.

### Sensitivity analysis

The sensitivity analyses were performed by sequential omitting individual studies to examine the influence of individual studies on the pooled ORs. The pooled ORs and corresponding 95% CIs were not significantly altered after excluding individual study in sequence (see Supplementary Fig. S1 online), suggesting that the overall results of our meta-analysis were stable and credible to some extent.

### Cumulative meta-analysis

Cumulative meta-analysis of the relationship between *WNK4* SNPs and hypertension were performed according to the year of publication. Regarding to C1155547T, as studies published in order, the statistical results had a trend to close to significant gradually and slightly. However, for G1156666A, G1155942T and C6749T polymorphisms, no obvious evolution rules were observed as records accumulated by year (see Supplementary Fig. S2 online).

## Discussion

Ten eligible articles contained 16 independent case-control studies with 6089 hypertensive cases and 4881 normotensive controls were included in this meta- analysis. To the best of our knowledge, this was the first meta-analysis to date regarding the association of *WNK4* gene polymorphisms with the risk of hypertension. The present study suggested a significant relationship between *WNK4* G1155942T polymorphism and hypertension in the allelic genetic model and dominant genetic model. Moreover, *WNK4* C6749T polymorphism was also found to be significantly associated with hypertension susceptibility under allele contrast, dominant genetic model and homozygous genetic model. Nevertheless, no significant association was identified between *WNK4* gene C1155547T and G1156666A polymorphisms and hypertension risk. Although the statistical bias could not be avoided completely, this study indicated that *WNK4* gene G1155942T and C6749T polymorphisms were associated with an increased risk of hypertension.

Human *WNK4* gene is mapped to chromosome 17 with 19 exons and spanning 16 kb of genomic DNA^12^. It has suggested that WNK4 is expressed almost exclusively in the kidney, and specifically localizes to the distal convoluted tubule (DCT) and the cortical collecting duct (CCD), the segment of the distal nephron involved in regulating the ion homestasis^11^,^36^. Previous studies both in vivo and in vitro suggested that *WNK4* modulated the balance between NaCl reabsorption and K^+^ secretion as a molecular switch via regulating the activities of the thiazide-sensitive Na-Cl cotransporter (NCC), the epithelia sodium channel (ENaC), the K^+^ channel (ROMK) and the paracellular Cl^−^ pathways principally^15^,^16^,^37^,^38^,^39^. Loss-of-function mutations of *WNK4* may cause increased NCC expression in the DCT and increase paracellular Cl^−^ permeability in the distal tubule and reduce the surface expression of ROMK channels^19^,^40^,^41^. The overactivity of NCC resulted of Na^+^ retention in the DCT contributing to development of hypertension. Consistently, the results of this meta-analysis confirmed the association of *WNK4* gene variation with the susceptibility of hypertension.

Wilson FH *et al.* firstly reported that mutations of *WNK1* and *WNK4* genes might be associated with PHA II, a rare autosomal dominant disease with the main performance of hypertension^14^. Since then, a series of animal experiments and epidemiologic studies concentrated on the association between *WNK1* and *WNK4* gene mutations and hypertension or blood pressure regulation. Studies by Putku *et al.*^21^ and Newhouse *et al.*^23^ separately demonstrated that polymorphisms in the *WNK1* gene were related with BP variation and replicated the association in their meta-analysis as well. However, no meta-analysis examining the association of *WNK4* gene mutations with the risk of hypertension existed. This study emerged under the circumstances in order to elucidate the role of *WNK4* gene polymorphisms in development of hypertension. According to the limited quantities of relevant eligible studies, we only selected four polymorphisms in *WNK4* gene (G1155942T, G1156666A, T1155547C, and C6749T) with at least 3 available relevant studies for further analyses. The mutations of *WNK4* gene G1662A, K1169E, Pro556Thr and Q565E were also suggested to be associated with blood pressure^42^,^43^,^44^. Better designed case-control or cohort studies involved in these gene loci may be retrieved to update our study much more comprehensively.

Though we tried our best to control the potential bias from statistical aspect and estimated the association between *WNK4* gene polymorphisms and hypertension as reliable as possible, some limitations of our study should be concerned. First, the included eligible studies without of large enough sample size limited the statistical power to some extent. Moreover, the quantities of each studied gene polymorphism were quite small and no sufficient data could be included for further analysis when we attempted to perform subgroup analyses. More studies with lager sample size are needed in the future to better interpret our results. Second, although the comprehensive literature search was performed in both English and Chinese database without language restriction, however, the *P*-value of Egger's test indicated the existence of publication bias for C1155547T polymorphism in association with hypertension. Third, the pathogenesis of hypertension is considered to be quite complicated involving gene-gene and gene-environment interactions. In this meta-analysis we tried to collect all the potentially relevant information such as BMI, gender distribution of the studied population, age and the level of serum lipid, however, we failed to assess the effect of gene-gene and gene-environment interactions. It has been reported that WNK kinases are upstream activators of ste20-related pralinealanine-rich kinase (SPAK) and ORS1 and the WNK-SPAK/OSR1 signaling pathway is very important in regulation of ion cotransporters and blood pressure^45^. The polymorphism of *STK39* gene encoding SPAK protein has been confirmed to be associated with hypertension by a meta-analysis recently^46^. Moreover, some other relevant gene polymorphisms including the calcium/calmodulin kinase IV (*CaMK4*) and G-protein-coupled receptor kinases (*GRKs*) are also demonstrated to related to regulation of blood pressure^47^,^48^.Together with intake of dietary salt, age, sex, many gene polymorphisms can play an influence on the regulation of blood pressure.

In conclusion, the current meta-analysis suggested that the G1155942T and C6749T polymorphisms of *WNK4* gene may increase the susceptibility of hypertension. Whereas, biological evidence was absent to support the association between *WNK4* C115547T and G1156666A polymorphisms and risk of hypertension. Hypertensive disease is one of the major causes of mortality and global burden of disease, timely recognition and effective intervention of hypertension can help control the hospital mortality of cardiovascular disease^49^,^50^. The results of our meta-analysis indicate that WNK4 might be a potential target for hypertension therapeutic intervention. However, due to the given limitations above, better designed studies with lager sample size and well-matched controls considering gene-gene and gene-environment interactions are needed in the future.

## Methods

### Strategy for literature search

A comprehensive literature search for relevant articles without language restrictions was performed through the following electronic database: PubMed, MEDLINE, Web of Science, China National Knowledge Infrastructure (CNKI), Wanfang Data and China Biology Medicine (CBM). To identify all possible studies as complete as possible, various keywords as “with-no-lysine kinase”, “*WNK*”, “variant”, “polymorphism”, “blood pressure”, “high blood pressure” and “hypertension” were used. We also manually searched the reference lists of the included studies to find additional eligible studies. The literature search was finally performed on March 20, 2014.

### Inclusion criteria

The studies were included in this meta-analysis if they comply with the following inclusion criteria: 1) evaluated the WNK4 (G1155942T, G1156666A, T1155547C, and C6749T) polymorphisms and hypertension; 2) used case-control or cohort design; 3) provided sufficient data of sample size, genotype distribution and allele frequencies or other information such as ORs with 95% CIs for statistical analysis; 4) diagnosis of hypertension patients based on the criteria of systolic blood pressure ≥ 140 mmHg or diastolic blood pressure ≥ 90 mmHg. If multi-studies reported the same or reduplicative data, we selected the latest published or the one with the largest sample size.

### Data extraction

Two authors (X. G. Guo and J Ding) extracted the following information from each qualified studies independently: first author's name, publication year, ethnicity of study population, study design, genotyping methods, source of the controls, sample size of cases and controls, the distribution of genotypes in cases and controls, and diagnostic criteria for hypertensive cases and normotensive controls. We also collected the baseline information of each study population, such as age, gender, body mass index (BMI), mean blood pressure of cases and controls, and serum concentration of total cholesterol (TC) and triglyceride (TG). If the inconsistent evaluations are encountered, the two authors reached a consensus by discussion or intervention of a third author.

### Statistical analysis

ORs and corresponding 95% CIs were used to evaluate the possible association between *WNK4* polymorphisms and hypertension. The heterogeneity among different studies was assessed by chi-square-based *Q*-tests^51^. And the value of *I*^*2*^ was used to quantify the effect of heterogeneity. If the *P*-value of the *Q-*test >0.10 or *I*^*2*^ <50%, we chose a fixed-effects model by Mantel-Haenszel method for the absence of significant heterogeneity. Otherwise, a random-effects model with the method of Dersimonian & Laird was used^52^,^53^.

We also performed sensitivity analysis by omitting an individual study each time to check whether any of these estimates can bias the overall estimate. Furthermore, cumulative meta-analysis was performed to estimate the influence on the subsequent studies by the first published study, and identified the evolution of pooled estimates as time goes by^54^.

Finally, publication bias was assessed by both Begg's test^55^ and Egger's regression test^56^. *P* < 0.05 was considered the existence of statistically significant publication bias. The HWE of controls was calculated using Pearson *χ*^2^ -test. The genotypes and allele frequencies of controls were considered in HWE if *P* > 0.05. All statistical analyses were performed using STATA 11.0 (StataCorp LP, College Station, Texas, USA).

## Author Contributions

Conceived and designed the experiments: G.X.G., D.J. Performed the experiments: X.T.M., J.W.Q., Y.X. Analyzed the data: S.Y.P., Z.F.R., Contributed reagents/materials/analysis tools: Z.J.H., Z.L.R., Wrote the paper: D.J., X.H., G.X.G.

## Supplementary Material

Supplementary Information Supporting Information

## Figures and Tables

**Table 1:** Characteristics of the eligible studies included in this meta-analysis

						Diagnostic criteria(mmHg)		Mean blood pressure(mm Hg)				Mean age (years)		Gender (Female(%))		BMI (kg/m2)		Cholesterol (mmol/L)		Triglycerides (mmol/L)	
								Cases		Controls											
Studied SNP and First author	Year	Country	Ethnicity	Genotyping method	Source of controls	Hypertensive	Normotensive	Systolic BP	Diastolic BP	Systolic BP	Diastolic BP	Cases	Controls	Cases	Controls	Cases	Controls	Cases	Controls	Cases	Controls
G1155942T (Ala 589 Ser;exon8)																					
Lv JY	2003	China	Caucasian	RT-PCR	NA	≥140/90	NA	NA	NA	NA	NA	NA	NA	NA	NA	NA	NA	NA	NA	NA	NA
Erlich PM *et al.*(Population 2)	2003	America	Negroid	PCR/Homogeneous MassEXTEND	HB	≥140/90^a,b^	<130/80^c^	NA	NA	NA	NA	NA	NA	NA	NA	NA	NA	NA	NA	NA	NA
Sun ZJ *et al.*	2009	China	Mongloid	PCR-RFLP	PB	≥140/90^a,b^	<140/90	156.12 ± 25.56	96.88 ± 11.92	113.23 ± 12.73	73.78 ± 8.99	51.45 (12.09)	49.50(13.97)	141(54.4)	136(57.9)	25.37(6.54)	22.02(3.57)	5.09(1.02)	4.39(1.08)	1.68(1.36)	1.06(0.86)
Lu M *et al.*	2009	China	Mongloid	TaqMan	PB	≥140/90^a,b^	≤ 120/80^d^	159.7 ± 22.1	96.7 ± 13.2	112.2 ± 10.2	71.5 ± 6.8	49.0(7.5)	48.7(10.8)	334(73.7)	291(64.0)	28.3 (5.3)	25.0(3.9)	4.7(1.1)	4.4(1.0)	1.7(1.1)	1.3(1.0)
Cao FF *et al.*	2010	China	Mongloid	TaqMan	PB	≥140/90^a,e^	<140/90^c^	NA	NA	NA	NA	48.86(10.47)	46.17(10.18)	309(54.9)	207(59.8)	26.36(4.20)	24.49(3.74)	5.17(1.52)	4.72(1.59)	1.39(0.94)	1.17(0.86)
G1156666A (intron10)																					
Erlich PM *et al.*(Population 1)	2003	America	Caucasian	PCR/Homogeneous MassEXTEND	HB	≥140/90^a,b^	<130/80^c^	NA	NA	NA	NA	NA	NA	NA	NA	NA	NA	NA	NA	NA	NA
Erlich PM *et al.*(Population 2)	2003	America	Negroid	PCR/Homogeneous MassEXTEND	HB	≥140/90^a,b^	<130/80^c^	NA	NA	NA	NA	NA	NA	NA	NA	NA	NA	NA	NA	NA	NA
Speirs HJ *et al.*	2004	Australia	Caucasian	Homogeneous MassEXTEND	PB	≥140/90	<130/90	173 ± 25	106 ± 15	119 ± 10	72 ± 8	52(12)	44(12)	112(61)	92(42)	27(5)	25(4)	4.9(0.1)	4.8(0.07)	1.9(0.1)	1.3(0.05)
Zhang LP *et al.*	2008	China	Mongloid	PCR-RFLP	PB	≥140/90^b^	<140/90	160.2 ± 18.6	96.1 ± 15.2	113.9 ± 13.2	75.0 ± 11.2	62.8(4.3)	61.8(6.6)	106(55.5)	101(58.4)	26.5(5.6)	24.4(4.1)	5.1(1.5)	4.8(1.9)	1.3(0.8)	1.1(0.6)
Lu M *et al*.	2009	China	Mongloid	TaqMan	PB	≥140/90^a,b^	≤120/80^d^	159.7 ± 22.1	96.7 ± 13.2	112.2 ± 10.2	71.5 ± 6.8	49.0(7.5)	48.7(10.8)	334(73.7)	291(64.0)	28.3(5.3)	25.0(3.9)	4.7(1.1)	4.4(1.0)	1.7(1.1)	1.3(1.0)
T1155547C (Ala 535 Ala; exon7)																					
Erlich PM *et al.*(Population 2)	2003	America	Negroid	PCR/Homogeneous MassEXTEND	HB	≥140/90^a,b^	<130/80^c^	NA	NA	NA	NA	NA	NA	NA	NA	NA	NA	NA	NA	NA	NA
Lu M *et al.*	2009	China	Mongloid	TaqMan	PB	≥140/90^a,b^	≤120/80^d^	159.7 ± 22.1	96.7 ± 13.2	112.2 ± 10.2	71.5 ± 6.8	49.0 (7.5)	48.7(10.8)	334(73.7)	291(64.0)	28.3(5.3)	25.0(3.9)	4.7(1.1)	4.4(1.0)	1.7(1.1)	1.3(1.0)
Cao FF *et al.*	2010	China	Mongloid	TaqMan	PB	≥140/90^a,e^	<140/90^c^	NA	NA	NA	NA	48.86(10.43)	46.31(10.14)	307(55.2)	206(60.4)	26.36(4.20)	24.47(3.78)	5.18(1.52)	4.72(1.60)	1.38(0.94)	1.17(0.86)
rs9916754 (C6749T; extron7)																					
Wang F	2008	China	Mongloid	TaqMan	PB	≥140/90^a,b,e^	<140/90^c^	165.71 ± 22.50	103.57 ± 12.00	118.43 ± 12.27	77.22 ± 6.99	48.86(10.43)	46.31(10.14)	307(55.2)	206(60.4)	26.36(4.20)	24.47(3.78)	5.18(1.52)	4.72(1.60)	1.38(0.94)	1.17(0.86)
Han Y *et al*.(The first study)	2011	China	Mongloid	PCR-RFLP	PB	≥140/90^a,b,e^	<130/85	154.2 ± 20.0	93.9 ± 11.2	119.0 ± 10.8	76.5 ± 6.9	58.3(8.4)	56.3(7.9)	533(65)	500(64.8)	25.7(3.5)	24.0(3.3)	5.46(1.11)	4.98(1.08)	NA	NA
Han Y *et al.*(The second study)	2011	China	Mongloid	PCR-RFLP	PB	≥140/90^a,b,e^	<130/85	NA	NA	NA	NA	NA	NA	NA	NA	NA	NA	NA	NA	NA	NA

NA: not available; PCR-RFLP: polymerase chain reaction-restriction fragment length polymorphism; RT-PCR: real-time PCR; HB: hospital based; PB: population based; BMI: Body Mass Index; a: and/or under antihypertensive treatment currently; b: and excluded secondary hypertension; c: and not being treated with antihypertensive medications; d: and not been diagnosed as hypertensive previously; e: and/or diagnosed as hypertensive in the past.*The continuous variables are expressed as means ± SD.

**Table 2:** Distribution of genotype and allele frequencies of each study included in this meta-analysis

Polymorphisms and First author	Year	Sample size		Genotypes						Allele frequencies (%)				HWE(*P*)
		Cases	Controls	Cases			Controls			Cases		Controls		
G1155942T (Ala 589 Ser;exon8)				GG	GT	TT	GG	GT	TT	G	T	G	T	
Lv JY	2003	896	451	864	30	2	447	4	0	98.1	1.9	99.6	0.4	0.92
Erlich PM *et al.*(Population 2)	2003	91	81	55	30	6	48	31	2	76.9	23.1	78.4	21.6	0.24
Sun ZJ *et al.*	2009	259	235	136	112	11	149	77	9	74.1	25.9	79.8	20.2	0.81
Lu M *et al*.	2009	451	454	0	24	427	0	31	423	2.7	97.3	3.4	96.6	0.45
Cao FF *et al*.	2010	563	346	526	36	1	338	8	0	96.6	3.4	98.8	1.2	0.83
G1156666A (intron10)				GG	GA	AA	GG	GA	AA	G	A	G	A	
Erlich PM *et al.*(Population 1)	2003	165	91	124	39	2	79	11	1	87	13	92.9	7.1	0.40
Erlich PM *et al.*(Population 2)	2003	113	94	109	4	0	90	4	0	98.2	1.8	97.9	2.1	0.83
Speirs HJ *et al.*	2004	184	219	152	29	3	176	43	0	90.5	9.5	90.2	9.8	0.11
Zhang LP *et al.*	2008	191	173	168	21	2	159	14	0	93.5	6.5	96	4	0.58
Lu M *et al.*	2009	452	452	415	36	1	399	53	0	95.8	4.2	94.1	5.9	0.19
C1155547T (Ala 535 Ala; exon7)				CC	CT	TT	CC	CT	TT	C	T	C	T	
Erlich PM *et al.*(Population 2)	2003	89	79	53	30	6	47	30	2	76.4	23.6	78.5	21.5	0.27
Lu M *et al.*	2009	451	454	0	24	427	0	31	423	2.7	97.3	3.4	96.6	0.45
Cao FF *et al.*	2010	556	341	518	37	1	333	8	0	96.5	3.5	98.8	1.2	0.83
rs9916754 (C6749T; extron7)				CC	CT	TT	CC	CT	TT	C	T	C	T	
Wang F	2008	556	341	518	37	1	333	8	0	96.5	3.5	98.8	1.2	0.83
Han Y *et al.*(The first study)	2011	801	767	672	120	9	701	65	1	91.4	8.6	95.6	4.4	0.69
Han Y *et al.*(The second study)	2011	271	303	228	41	2	272	30	1	91.7	8.3	94.7	5.3	0.86

HWE (*P*): the *P*-values of the Hardy-Weinberg equilibrium test of control group.

**Table 3:** The main results of the meta-analysis of the association between the *WNK4* variant

		Sample size							
Studied Polymorphisms	*n*	case	control	Genetic model	Statistical model	OR(95%CI)	*P*_z_	I^2^ (%)	*P*_heterogeneity_
C1155547T	3	1096	874	Allele contrast	Random model	1.54(0.90,2.63)	0.11	58.4	0.09
				Dominant	Random model	1.70(0.57,5.13)*	0.35	79.9	0.03
				Homozygous	Fixed model	2.49(0.57,10.78)*	0.22	0	0.86
G1156666A	5	1095	1029	Allele contrast	Random model	1.12(0.74,1.69)	0.6	54.8	0.07
				Dominant	Random model	1.08(0.68,1.71)	0.74	59	0.05
				Homozygous	Fixed model	3.40(0.86,13.54)^#^	0.08	0	0.8
G1155942T	5	2260	1567	Allele contrast	Random model		0.01	57.1	0.05
				Dominant	Random model		0.03	64.7	0.04
				Homozygous	Fixed model	1.67(0.80,3.49)[Fn t3-fn1]	0.18	0	0.9
C6749T	3	1628	1411	Allele contrast	Fixed model		0	0	0.37
				Dominant	Fixed model		0	0	0.42
				Homozygous	Fixed model		0.02	0	0.59

*: for C1155547T, the study by Lu M *et al*. 2009 was excluded under the dominant and homozygous genetic model because of the absence of wild homozygote(CC) in both case and control group. #: for G1156666A, the study by Erlich PM *et al*. (population 2) 2003 was excluded under the homozygous genetic model because of the absence of mutational homozygote (AA) in both case and control group. : for G1155942T, the study by Lu M *et al.* 2009 was excluded under the dominant and homozygous genetic model because of the absence of wild homozygote(GG) in both case and control group. 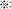: OR had statistical significance with corresponding 95% CI greater than 1.
