# Supplementary material for: Comprehensive Assessment of the Association of WNK4 Polymorphisms with Hypertension: Evidence from a Meta-Analysis
Source: Sci Rep. 2014 Sep 30;4:6507. doi: 10.1038/srep06507 (PMC4195396; doi:10.1038/srep06507)
Supplement: Supplementary Information — Supporting Information [file srep06507-s1.pdf]

Supporting Information for:

Comprehensive Assessment of the Association of *WNK4* Polymorphisms with Hypertension: Evidence from a Meta-Analysis

Xiao-gang GUO[1,] Jie DING[1], Hui XU[1,2], Tian-ming Xuan[1], Wei-quan Jin[1], Xiang YIN[1], Yun-peng Shang[1], Fu-rong ZHANG[1], Jian-hua ZHU[1] & Liang-rong ZHENG[1]

[1]Department of Cardiology, the First Affiliated Hospital, School of Medicine, Zhejiang University, Hangzhou 310003, China; [2]Xiuzhou District, Gaozhao Street Community Health Service Center, Jiaxing 314031, China

1. Sensitivity Analysis

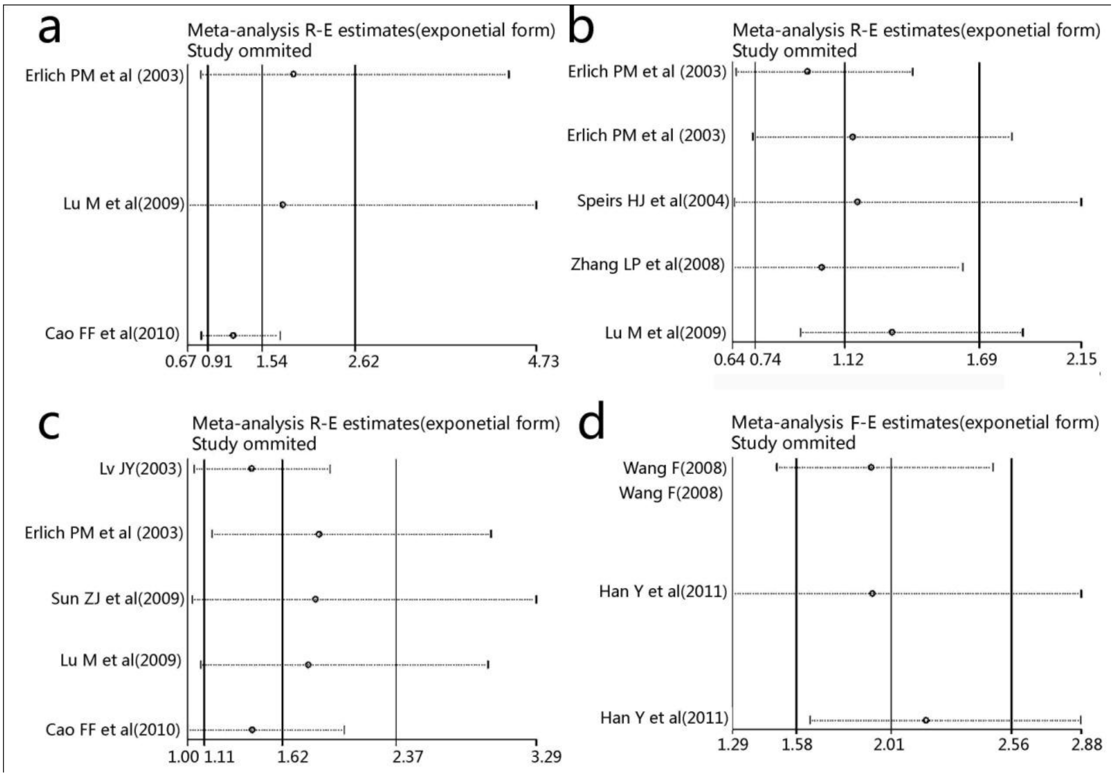

Supplementary Fig. S1| Sensitivity analysis to examine the influence of individual studies on the pooled Ors under allele contrast. The circles represent the pooled ORs when the given named study on the left is omitted, and the dotted lines indicate the 95% CIs. a: C1155547T polymorphism and hypertension; b: for G1156666A polymorphism and hypertension; c: G1155942T polymorphism and hypertension; d: C6749T polymorphism and hypertension; R-E: random effects; F-E: fixed effects.

2. Cumulative meta-analysis

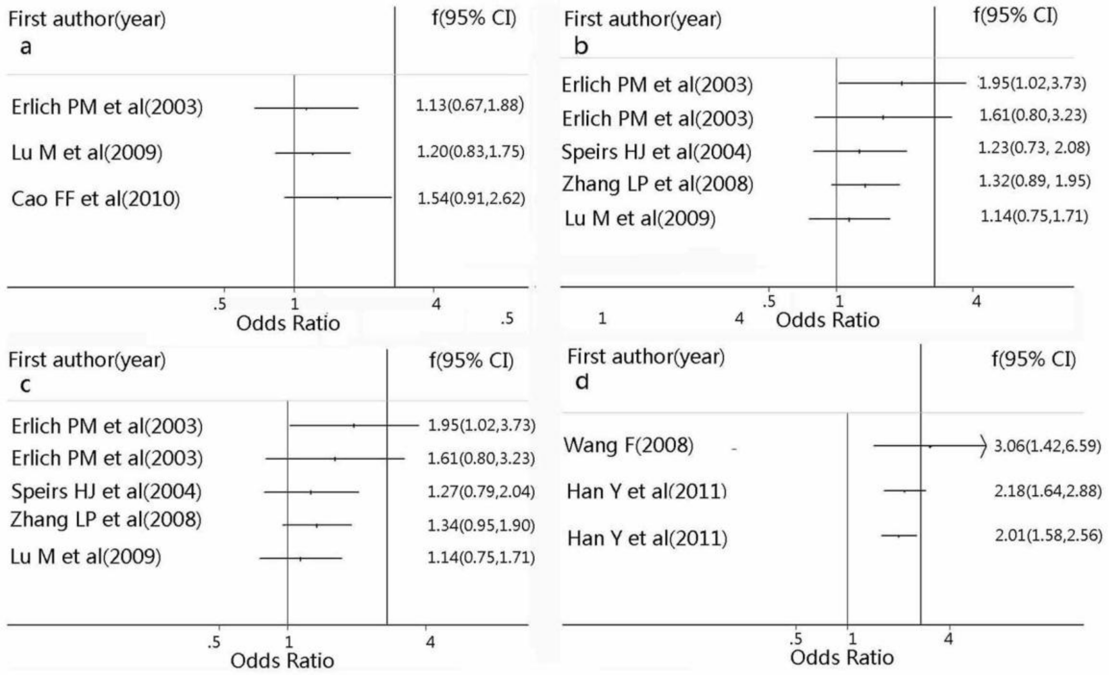

Supplementary Fig. S2| Cumulative meta-analysis of the association between *WNK4* SNPs and hypertension under allele contrasts. a: C1155547T polymorphism; b: G1156666A polymorphism; c: G1155942T polymorphism; d: C6749T polymorphism.
